# Influence of Genotype and Slaughter Age on the Content of Selected Minerals and Fatty Acids in the Longissimus Thoracis Muscle of Crossbred Bulls

**DOI:** 10.3390/ani10112004

**Published:** 2020-10-30

**Authors:** Martyna Momot, Zenon Nogalski, Paulina Pogorzelska-Przybyłek, Monika Sobczuk-Szul

**Affiliations:** Department of Cattle Breeding and Milk Evaluation, Faculty of Animal Bioengineering, University of Warmia and Mazury in Olsztyn, ul. Oczapowskiego 5/137, 10-719 Olsztyn, Poland; zenon.nogalski@uwm.edu.pl (Z.N.); paulina.pogorzelska@uwm.edu.pl (P.P.-P.); monika.sobczuk@uwm.edu.pl (M.S.-S.)

**Keywords:** beef, crossbred bulls, minerals, fatty acids

## Abstract

**Simple Summary:**

In some European countries, including Poland, beef production is based mostly on dairy cattle herds. Beef quality can be improved by the commercial crossbreeding of dairy cows with beef bulls. The offspring are characterized by higher fattening performance and more desirable carcass characteristics. The experiment was performed on 72 bulls produced by crossing Polish Holstein-Friesian (PHF) cows with bulls of three most popular beef breeds: Hereford (HH), Limousin (LM), and Charolais (CH), to determine the effects of genotype and age at slaughter on the content of selected minerals (K, Na, Mg, Zn, and Fe) and fatty acid profile in beef. The meat of PHF × LM crosses had a lower content of K and Mg, compared with the remaining crossbred bulls. The Fe content per kg of meat was higher in bulls slaughtered at 21 months of age than in those slaughtered at 15 months of age. The best sire breed for crossing with dairy cows cannot be clearly indicated based on the present findings. However, the results of this study suggest that bulls should be slaughtered at 21 months of age to achieve the optimal values of most analyzed traits and parameters, in particular the fatty acid profile.

**Abstract:**

The aim of this study was to determine the effects of genotype and slaughter age on the mineral content and fatty acid profile of beef. The experiment was performed on 72 crossbred bulls produced by crossing Polish Holstein-Friesian (PHF) cows with bulls of three beef breeds: Hereford (HH), Limousin (LM), and Charolais (CH), slaughtered at 15, 18, and 21 months of age. Samples of the longissimus thoracis muscle were collected to determine their mineral (potassium—K, sodium—Na, magnesium—Mg, zinc—Zn, and iron—Fe) and fatty acid composition. The meat of PHF × LM crosses had a lower (*p* ≤ 0.01) content of K and Mg, compared with the remaining crossbred bulls. The Fe content per kg of meat was 4 mg higher (*p* ≤ 0.01) in bulls slaughtered at 21 months of age than in those slaughtered at 15 months of age. The content of monounsaturated fatty acids (MUFAs) in intramuscular fat was 2.77% higher in bulls slaughtered at 21 months of age than in those slaughtered at 15 months of age. The n-6/n-3 polyunsaturated fatty acids (PUFA) ratio did not exceed 4.0, and it was most desirable in PHF × LM crosses (2.84) and in the oldest bulls (2.92).

## 1. Introduction

Red meat, including beef, is an important source of highly biologically active proteins, fat, vitamins, and minerals (in particular, iron—Fe and zinc—Zn). Fat is the richest source of dietary energy, and it supplies essential nutrients such as fat-soluble vitamins and fatty acids [1]. Bovine fat contains fatty acids that deliver health benefits to humans. n-3 and n-6 polyunsaturated fatty acids (PUFAs) must be derived from the diet because they cannot be synthesized de novo by the human body or other monogastric mammals. n-3 and n-6 PUFAs act as carriers of fat-soluble vitamins (A, D, E, and K) and play a key role in the immune response. Linoleic acid, a member of the n-6 fatty acid family, contributes to lowering serum cholesterol levels and reducing the risk of cardiovascular disease. It is an important structural constituent of cell membranes, a function thought to be related to dermatitis in infants [2]. Eicosapentaenoic acid (EPA) and docosahexaenoic acid (DHA), members of the n-3 fatty acid family, help prevent cardiovascular disease and arrhythmia, lower plasma triacylglycerol levels, decrease blood pressure, reduce platelet aggregation, and exert anti-inflammatory effects [3,4]. Conjugated linoleic acid (CLA) is a group of isomers of linoleic acid found mostly in the meat and dairy products derived from ruminants. The major CLA isomers are cis-9 trans-11, and trans-10 cis-12. Research has shown that CLA has immunomodulatory, antimutagenic, anticarcinogenic, antidiabetic (type II diabetes), and antihypertensive properties. It can also play a role in preventing obesity, atherosclerosis, lifestyle diseases, and the metabolic syndrome [5,6,7]. Vaccenic acid is a trans fatty acid, but it is believed to provide health benefits to humans. It is the predominant trans fatty acid found in red meat, and it does not affect the blood levels of total or low-density lipoprotein (LDL) cholesterol [8]. Zn is important for the immune system; it participates in the regulation of cell growth and proliferation, is a component of numerous metalloenzymes, and contributes to antioxidant defense. The major sources of Zn in the human diet are red meat, seafood, poultry, cereals, dairy products, legumes, and vegetables [9]. Red meat is a rich source of readily bioavailable Zn, whereas cereals have a high content of phytic acid, a strong inhibitor of Zn absorption [10]. Fe plays an important role as an oxygen carrier in hemoglobin (in red blood cells) and myoglobin (in muscle cells), and it is involved in numerous metabolic processes [1]. Heme Fe found in meat is more bioavailable than non-heme Fe found in plant-based foods [9]. Magnesium (Mg) has a strong relation with the immune system, it is involved in both non-specific and specific immune responses, also referred to as innate and acquired immune responses, respectively [11].

Mineral concentrations in meat change depending on the diet [12], genotype and age of an animal [13], muscle type [14,15,16], and geographic conditions [17,18]. The fatty acid composition of beef is mostly affected by the breed, age, and sex category of an animal [19,20], as well as dietary factors [21,22]. The differences in the fatty acid profile between cattle breeds result from the differences in gene expression and the activity of enzymes that participate in fatty acid synthesis [23]. As animals grow older, the content of subcutaneous tissue and muscular fat increases, whereas the ratio of PUFAs to saturated fatty acids (SFAs) decreases [24]. Furthermore, pre-slaughter stressors can impact on ruminant carcass and meat quality. Loudon et al. [25] observed that plasma creatine kinase was positively correlated with ultimate pH, indicating that cattle with higher muscle trauma had a higher rate of glycogen depletion pre-slaughter.

In Poland, beef production is based mostly on dairy cattle herds. Beef quality can be improved by the commercial crossbreeding of dairy cows with beef bulls. The offspring are characterized by higher fattening performance and more desirable carcass characteristics [26]. The most popular beef cattle breeds in Poland are Hereford (HH), Limousin (LM), and Charolais (CH), and bulls of these breeds are usually used as the paternal component for crossing with dairy cows. The aim of this study was to determine the effects of genotype and age at slaughter on the content of selected minerals and fatty acid profile in meat from crossbred bulls produced by crossing Polish Holstein-Friesian (PHF) cows with HH, LM, and CH bulls.

## 2. Materials and Methods

### 2.1. Animals

The study was conducted upon the approval of the Ethics Committee of the University of Warmia and Mazury (decision no. 121/2010). The experimental material comprised 72 crossbred bulls produced by crossing PHF cows with HH, LM and CH bulls. Crossbred calves were purchased at 2–3 weeks of age. The animals underwent a seven-day quarantine period and were raised until six months of age. They were fed milk replacer supplemented with hay and concentrate, followed by grass silage. The fattening period began when the calves were 181 days old. The animals were fattened in a semi-intensive system, and they were fed ad libitum a total mixed ration (TMR) composed of grass silage and concentrate (Table 1 and Table 2).

The concentrate to silage ratio, on a dry matter (DM) basis, was 25:75 (Table 2). When the bulls reached a body weight (BW) of 300 kg, concentrate composition was modified to account for age-related changes in the dietary protein to energy ratio. Bulls with BW below 300 kg were fed a TMR containing concentrate I (triticale, 71%; rapeseed meal, 25%; premix (Cargill Poland Ltd., Warsaw, Poland), 4%), and bulls with BW above 300 kg were fed a TMR containing concentrate II (triticale, 77%; rapeseed meal, 19%; premix, 4%). The animals were fattened in a free-stall barn until 15, 18, and 21 months of age.

### 2.2. Carcass Quality

At the end of the fattening period, the bulls were transported to a meat processing plant, where they were fasted for 24 h and slaughtered. The animals were weighed before slaughter, stunned, slaughtered, bled, dressed, and split into two sides that were then chilled for 96 h at +4 °C. Electrical stimulation was not applied to the carcasses. Half-carcasses were weighed within an accuracy of 0.5 kg, using an automatic in-line scale. Carcass conformation and fatness were evaluated by a trained grader, based on the EUROP system criteria [27]. Dressing percentage (percentage ratio of carcass weight to slaughter weight) was calculated. After 96 h of carcass chilling, samples weighing approximately 200 g were collected from the longissimus thoracis (LT) muscle between the 11th and 13th thoracic vertebrae. Vacuum-packaged samples were transported (+4 °C) to the laboratory. Slaughter and post-slaughter processing were carried out in accordance with Council Regulation (EC) No 1099/2009 of 24 September 2009 on the protection of animals at the time of slaughter [28]. During the experiment, the animals remained under veterinary care and were regularly visited by an animal care specialist.

### 2.3. Mineral Content of Meat

In order to determine the content of selected minerals, samples of the LT muscle were homogenized, and approximately 0.5 g of the muscle tissue was wet mineralized with 7 mL of 65% Suprapur nitric acid (Merck, Darmstadt, Germany) in the MARS Xpress 5 microwave digestion system (CEM Corporation, Matthews, NC, USA). Each mineralization procedure involved two blank samples and two samples of Standard Reference Material^®^ 1577c Bovine Liver (NIST, Gaithersburg, MD, USA). All samples were mineralized and analyzed in duplicate. The mineralized samples were cooled and quantitatively transferred to 25 cm^3^ volumetric flasks. The content of selected minerals (potassium—K, sodium—Na, magnesium—Mg, zinc—Zn, and iron—Fe) in beef samples was determined by flame atomic absorption spectrometry (flame AAS) with the use of the AA240FS Fast Sequential Atomic Absorption Spectrometer (Varian, Palo Alto, CA, USA) [29,30].

### 2.4. Fatty Acid Profile

Intramuscular fat (IMF) was extracted from ground meat samples by the Soxhlet method [31] using the B-811 extraction system (Büchi, Flawil, Switzerland), with n-hexane as the solvent (POCH, Gliwice, Poland). Fatty acid methyl esters (FAMEs) were obtained by methylation in a methanol–chloroform–H_2_SO_4_ mixture (POCH, Gliwice, Poland; STANLAB, Lublin, Poland; STANLAB, Lublin, Poland, respectively) according to the modified Peisker method [32] and Standard PN-EN ISO 5509 [33]. The content of 33 fatty acids in IMF was determined with the use of the CP-3800 gas chromatograph (Varian, Palo Alto, CA, USA) equipped with a split/splitless injector and a flame-ionization detector (FID). Samples (1 µL) of FAMEs were placed on the Varian Capillary Column Select™ FAME (length: 100 m; inner diameter: 0.25 mm) using a Hamilton syringe and the CP-8410 autosampler (Varian, Palo Alto, CA, USA). Helium was used as the carrier gas. Injector temperature was 260 °C. The total time of a single analysis was 68 min. Fatty acids were identified by comparing their retention times with the retention times of individual reference standards and their mixtures, purchased from Supelco (Sigma Aldrich, Bellefonte, PA, USA). Analyses of the samples and reference standards were performed under identical separation conditions. The instrument was controlled, and the data were acquired and processed using the GALAXIE Chromatography Data System. Fatty acids were divided into the following categories; saturated fatty acids (SFAs), unsaturated fatty acids (UFAs), including monounsaturated fatty acids (MUFAs), and polyunsaturated fatty acids (PUFAs), as well as n-6 and n-3 PUFAs. The following ratios were calculated; MUFA/SFA, PUFA/SFA, and n-6/n-3 PUFA.

### 2.5. Statistical Analysis

The data were analyzed statistically using Statistica version 13.3 software [34]. The effects of genotype (PHF × HH, PHF × LM and PHF × CH) and slaughter age (15, 18, and 21 months) on the fatty acid profile and mineral content of beef were determined by the least squares method using the formula

Y_ijk_ = μ + A_i_ + B_j_ + (AB)_ij_ + e_ijk_ (1)

where Y_ijk_ is the value of the analyzed parameter, μ is population mean, A_i_ is the effect of genotype (PHF × HH, PHF × LM, PHF × CH), B_j_ is the effect of slaughter age (15, 18, 21), (AB)_ij_ is the genotype × slaughter age interaction, and e_ijk_ is random error. The differences between means were estimated by Tukey’s test.

## 3. Results and Discussion

As expected, older animals were characterized by higher BW at slaughter (*p* ≤ 0.01) (Table 3). The final BW of crossbred bulls was not affected by sire breed although the beef breeds used for crossing (HH, LM, and CH) represent different maturity types. Genotype had a significant effect on carcass dressing percentage and fat content. The dressing percentage of PHF × LM crosses was 1.79% higher (*p* ≤ 0.01) than that of PHF × CH crosses. Dressing percentage is a key indicator of carcass quality in cattle. The LM breed is known for its high carcass quality, which was confirmed by Chambaz et al. [35] in whose study, dressing percentage was 4.4% higher in LM steers than in CH steers. In the present experiment, PHF × HH crosses received the highest fatness score, and the difference relative to PHF × LM crosses was statistically significant (*p* ≤ 0.05). Carcass dressing percentage, conformation, and fatness were significantly affected by age at slaughter. Dressing percentage was higher in heavier animals, which could result from their higher carcass fatness. In a previous study investigating young Holstein-Friesian bulls [36], higher BW at slaughter was associated with higher carcass fat content.

The mineral composition of the LT muscle of crossbred bulls is presented in Table 4. Genotype had a significant influence on the concentrations of K, Mg, and Fe. Meat from PHF × CH and PHF × HH crosses had a higher (*p* ≤ 0.01) content of K and Mg than meat from PHF × LM crosses. Fe content was higher (*p* ≤ 0.05) in meat from PHF × CH crosses than in meat from PHF × LM crosses. The content of Zn and Fe in the LT muscle was affected by age at slaughter. The meat of animals slaughtered at 18 months of age had the highest (*p* ≤ 0.05) Zn content (63.9 mg/kg of fresh meat), whereas the meat of the oldest bulls had the highest Fe content (19 mg/kg of fresh meat). The effect of cattle breed on the K and Mg content of beef was also noted by Pilarczyk [37] and Domaradzki et al. [38]. In the study by Pilarczyk [37], the Fe content of meat was also affected by the genotype. Pilarczyk [37] found that meat from CH bulls was more abundant in K than meat from HH and Simmental bulls. K concentrations in meat samples collected from LM, Red Angus, Salers, CH, HH, and Simmental bulls [37,39] were considerably lower (2377.0–2506.1 mg/kg of fresh meat) than those noted in the current experiment (4750.2–4979.8 mg/kg of fresh meat). The K content of meat from Angus bulls, reported by Mateescu et al. [40], was also lower than the values determined in the present study. One-hundred grams of meat supplied 5.7–6% of the adequate intake for Na for adults. In the case of K, consumption of 100 g of cooked beef supplied 19.8–20.8% of the daily standard for men and 25.9–27.1% for women [41]. In contrast to our findings, Doornenbal and Murray [42], and Kotula and Lusby [43] demonstrated that age at slaughter affected K levels in beef. However, Doornenbal and Murray [42] observed changes in K concentrations in cattle aged 3 to 12 years. Kotula and Lusby [43] reported that the K content of meat was influenced by age at slaughter in steers aged 1 to 6 years, and it decreased from 392 mg/100 g of wet tissue in 24-month-old animals to 332 mg/100 g of wet tissue in 72-month-old animals. However, the cited authors [43] noted no significant changes in the concentrations of K, Na, Mg, or Zn in Angus steers aged 12 and 24 months.

In the current study, neither breed nor age at slaughter exerted significant effects on the Na content of beef. The lowest Mg content of meat was noted in meat from PHF × LM crosses and bulls slaughtered at 18 months of age (183.0 mg/kg of fresh meat and 199.3 mg/kg of fresh meat, respectively). In the remaining groups, Mg concentrations exceeded 200 mg/kg of fresh meat. Consumption of 100 g of cooked beef satisfied 8.1–9.6% and 6.2–7.3% of the recommended daily allowance (RDA) for Mg for women and men, respectively [41]. Pilarczyk [39] found that meat from HH bulls had lower Mg content than meat from CH bulls, which is consistent with the present results. Regardless of cattle breed, Pilarczyk [37,39] noted higher average Mg concentration in beef (263.4 mg/kg of fresh meat), compared with our experiment. In the present study, the Zn content of the LT muscle ranged from 38.5 to 63.9 mg/kg of fresh meat. The present study shows that cooked beef is an important source of Zn, as an edible portion of 100 g met satisfies 58.3–96.8% of their daily requirement for males and 80.2–133.1% for females [41]. Meat from bulls slaughtered at 18 months of age and PHF × HH crosses was the richest source of Mg. Beef was a rich source of Fe and the consumption of 100 g of cooked meat satisfies 13.2–16.7% and 29.7–37.6% of the RDA for females and males, respectively [41]. Littledike et al. [44] compared mineral concentrations in nine beef cattle breeds and found that Fe content was lowest in LM and Red Poll cows. In the current study, Fe content ranged from 15.0 to 19.0 mg/kg of fresh meat, and it was lowest in PHF × LM crosses. In the experiments conducted by Cabrera et al. [14] and Ramos et al. [45], Fe content ranged from 30 to 45 mg/kg of wet tissue and from 35 to 40 mg/kg of wet tissue in HH and Braford steers, respectively. In the present study, age at slaughter had a significant effect on Fe concentration in beef, which increased with age. Giuffrida-Mendoza et al. [13] demonstrated that Fe levels remained unchanged in samples collected from water buffalo and Zebu-influenced cattle aged 17 and 19 months, but decreased significantly at 24 months of age. According to Duckett et al. [46], differences in the mineral composition of beef can be attributed to differences between genetic groups, feed types and finishing systems. According to García-Vaquero et al. [47], in animals characterized by an adequate mineral status, essential trace element concentrations in muscle tissue depend on individual muscle metabolism.

The concentrations of fatty acids, major fatty acid groups, and ratios in the IMF of crossbred bulls are presented in Table 5, Table 6 and Table 7. The IMF content of the LT muscle was affected by age at slaughter and, as expected, it increased with age up to 1.80%. A significant difference in IMF content was noted between bulls slaughtered at 15 and 18 months of age. According to many authors [48,49], beef from LM cattle is lean. In the present study, IMF content was lowest in PHF × LM crosses, and highest in PHF × HH crosses, which resulted from physiological differences between early- and late-maturing breeds. Such a low IMF content of meat was affected by diets based on grass silage supplemented with concentrate. In the current experiment, age at slaughter had a significant effect on the concentrations of SFAs, UFAs and the MUFA/SFA ratio (*p* ≤ 0.01), and on the concentrations of MUFAs (*p* ≤ 0.05) in the LT muscle of bulls. The IMF of animals slaughtered at 21 months of age had a significantly higher content of UFAs and MUFAs, compared with those slaughtered at 15 months of age. Age at slaughter had a significant influence on the percentage share of the following fatty acids in IMF: oleic, C15:0 anteiso (*p* ≤ 0.01), capric, lauric, pentadecanoic, margaric, stearic, and gadoleic (*p* ≤ 0.05). The IMF of bulls slaughtered at 15 months of age had the highest concentrations of capric acid and lauric acid, whereas the IMF of the oldest bulls had the lowest content of the following fatty acids: capric, lauric, C15:0 anteiso, pentadecanoic, margaric and stearic. The IMF of the oldest bulls had the highest proportion of UFAs, including oleic acid (a significant difference was noted between animals slaughtered at 21 and 15 months of age) and gadoleic acid.

The n-6/n-3 PUFA ratio was affected by genotype and age at slaughter. A significant (*p* ≤ 0.01) difference was observed between PHF × LM crosses (2.84) and PHF × CH crosses (3.3). The most desirable n-6/n-3 PUFA ratio was noted in bulls slaughtered at 21 months of age (2.92), with a significant (*p* ≤ 0.05) difference relative to those slaughtered at 15 months of age.

In the present study, UFAs were the predominant fatty acids in the IMF of bulls, and their proportion was lower than that of SFAs only in bulls slaughtered at 15 months of age. The concentrations of UFAs and MUFAs increased, whereas the proportion of SFAs decreased with age. In the fatty acid profile of IMF, PUFAs accounted for 6.73–7.47%, and their proportion was highest in PHF × CH crosses and in bulls slaughtered at 18 months of age. The most abundant fatty acids were oleic acid (34.17–36.82%), palmitic acid (25.17–26.24%), and stearic acid (17.35–19.38%). The predominant SFAs in beef are palmitic acid, stearic acid, and myristic acid. Lauric acid, myristic acid, and palmitic acid are associated with elevated plasma total and LDL cholesterol concentrations, which are not increased by stearic acid [4]. Elaidic acid, the major industrially produced trans fat, can exert adverse effects on human health by negatively affecting cholesterol metabolism [8]. In the current experiment, its concentration ranged from 0.470% to 0.523%. The minimum recommended PUFA/SFA ratio in the human diet is 0.45 [50]. Its values noted in this study were considerably lower (0.137–0.156), due to too low PUFAs concentrations in the analyzed samples. According to the British Department of Health [50], the n-6/n-3 PUFA ratio should not exceed 4.0, and it was determined at 2.84–3.30 in this study. A high dietary n-6/n-3 PUFA ratio is considered a risk factor for selected lifestyle diseases. The n-6/n-3 PUFA ratio in the modern Western diet is much too high, reaching 10–15:1, whereas its optimal range is 2–4:1 [3]. In comparison with the present study, De Freitas et al. [51] noted a lower proportion of SFAs (46.42%), a similar proportion of MUFAs (43.38%), and a considerably higher proportion of PUFAs (10.05%) and the n-6/n-3 PUFA ratio (4.87) in the meat of HH steers. Contrary to our findings, Ugarković et al. [52] found lower SFAs concentrations and higher MUFAs concentrations in beef from HH cattle, compared with the CH breed. The following fatty acids analyzed in this study deliver health benefits: vaccenic acid, oleic acid, linoleic acid, CLA, α-linolenic acid, arachidonic acid, EPA, docosapentaenoic acid (DPA), and DHA. The oleic acid content of the LT muscle increased with age. The concentrations of linoleic acid, CLA, α-linolenic acid, arachidonic acid, EPA, and DPA were highest in PHF × CH crosses, whereas PHF × LM crosses were characterized by the highest levels of vaccenic acid and DHA. Cis-9 trans-11 is the most prevalent CLA isomer, accounting for 80–90% of the total CLA in ruminant tissues [5]. In the current experiment, its content ranged from 0.278% to 0.302%.

## 4. Conclusions

Different beef breed sires used for commercial crossing affected the content of K, Mg, and Fe in beef. The LT muscle of PHF × LM crosses had the lowest content of K, Mg, and Fe. Bulls slaughtered at a later age were characterized by higher Fe content and higher concentrations of MUFAs, in particular, oleic acid and gadoleic acid. The n-6/n-3 PUFA ratio did not exceed the nutritionally desirable value of 4.0, and it was lowest in PHF × LM crosses and in bulls slaughtered at 21 months of age. The best sire breed for crossing with dairy cows cannot be clearly indicated based on the present findings. However, the results of this study suggest that bulls should be slaughtered at 21 months of age to achieve the optimal values of most analyzed traits and parameters, in particular the fatty acid profile.

## Figures and Tables

**Table 1:** Chemical composition and nutritional value of diets (mean ± standard error of the mean).

Specification	Silage	Triticale	Rapeseed Meal	Concentrate I	Concentrate II
DM ^1^ (g)	397 ± 0.91	881 ± 0.96	887 ± 0.68	883.9 ± 1.12	885.5 ± 1.27
On DM basis (g∙kg^−1^)					
Organic matter	920 ± 2.46	981 ± 0.95	927 ± 1.05	928 ± 1.14	921 ± 1.39
Crude protein	141 ± 1.48	133 ± 1.32	388 ± 1.39	191 ± 1.15	175 ± 1.13
NDF ^2^	569 ± 5.31	193 ± 1.63	310 ± 0.68	214 ± 1.21	205 ± 1.92
ADF ^3^	387 ± 0.92	44 ± 0.65	228 ± 0.67	88 ± 1.85	76 ± 1.22
UFV ^4^	0.80 ± 0.03	1.21 ± 0.33	1.01 ± 0.05	1.11 ± 0.03	1.12 ± 0.02
PDIN ^5^	82.2 ± 1.64	89 ± 0.38	259 ± 0.57	127.2 ± 0.41	116.4 ± 1.23
PDIE ^6^	69.5 ± 0.58	109 ± 1.03	163 ± 1.56	118.6 ± 1.26	113.1 ± 1.71

^1^ DM—Dry matter. ^2^ NDF—Neutral detergent fiber. ^3^ ADF—Acid detergent fiber. ^4^ UFV—Meat Production Units. ^5^ PDIN—Protein digested in the small intestine when rumen-fermentable nitrogen is limiting. ^6^ PDIE—Protein digested in the small intestine when rumen-fermentable energy is limiting. N = 9 for silage. N = 5 for triticale and rapeseed meal. N = 7 for concentrates. Fermentation characteristics of silage: pH 4.8 ± 0.3; lactic acid, 5.4 ± 2.0% dry matter; volatile fatty acids, 2.7 ± 0.5% dry matter; water-soluble carbohydrates; 8.2 ± 4.8% dry matter; NNH3%N, 10.3 ± 6.7; true protein 51.8 ± 4.6% crude protein.

**Table 2:** Chemical composition and nutritional value of experimental total mixed rations (mean ± standard error of the mean).

Specification	BW ^1^ < 300 kg	BW ^1^ > 300 kg
Grass silage (% DM in diets)	75	75
Concentrate I (% DM in diets)	25	
Concentrate II (% DM in diets)		25
DM ^2^ (g)	509.6 ± 0.78	514.6 ± 0.95
On DM basis (g∙kg^−1^)		
Crude protein	153 ± 1.23	149 ± 1.18
NDF ^3^	472 ± 1.56	463 ± 3.01
ADF ^4^	311 ± 0.89	295 ± 2.09
UFV ^5^	0.88 ± 0.09	0.88 ± 0.03
PDIN ^6^	93.6 ± 0.45	90.8 ± 1.03
PDIE ^7^	81.6 ± 1.08	80.6 ± 1.63

^1^ BW—Body weight. ^2^ DM—Dry matter. ^3^ NDF—Neutral detergent fiber. ^4^ ADF—Acid detergent fiber. ^5^ UFV—Meat Production Units. ^6^ PDIN—Protein digested in the small intestine when rumen-fermentable nitrogen is limiting. ^7^ PDIE—Protein digested in the small intestine when rumen-fermentable energy is limiting.

**Table 3:** Effects of genotype and slaughter age on carcass quality in crossbred bulls.

Specification	Crossbred Bulls (C)			Slaughter Age (Months) (SA)			SE ^8^	*p*-Value		
	PHF ^1^ × HH ^2^	PHF ^1^ × LM ^3^	PHF ^1^ × CH ^4^	15	18	21		C	SA	C × SA
BW ^5^ before slaughter (kg)	505.0	497.7	513.5	424.3 ^A^	494.8 ^B^	596.9 ^C^	10.96	0.651	0.000	0.076
Dressing percentage (%)	58.06	59.42 ^A^	57.63 ^B^	58.01 ^a^	57.88 ^a^	59.22 ^b^	0.26	0.008	0.044	0.351
Conformation score ^6^ (pts)	7.8	7.7	7.3	7.1 ^A^	7.2 ^A^	8.4 ^B^	0.19	0.472	0.007	0.884
Fatness score ^7^ (pts)	5.5 ^a^	4.2 ^b^	4.9	3.9 ^A^	4.9	5.8 ^B^	0.21	0.018	0.000	0.355

^1^ PHF—Polish Holstein-Friesian. ^2^ HH—Hereford. ^3^ LM—Limousin. ^4^ CH—Charolais. ^5^ BW—Body weight. ^6^ EUROP conformation: 1 to 15, with 1 = very lean, 15 = muscled outstanding. ^7^ EUROP degree of fat cover: 1 to 15, with 1 = very low, 15 = very fat. ^8^ SE—standard error. Means followed by different superscript letters differ within rows (within the factor): A, B, C (*p* ≤ 0.01); a, b (*p* ≤ 0.05).

**Table 4:** Mineral (mg/kg of fresh meat) content of the longissimus thoracis muscle of crossbred bulls.

Minerals	Crossbred Bulls (C)			Slaughter Age (Months) (SA)			SE ^10^	*p*-Value		
	PHF ^1^ × HH ^2^	PHF ^1^ × LM ^3^	PHF ^1^ × CH ^4^	15	18	21		C	SA	C × SA
K ^5^	4963.4 ^A^	4750.2 ^B^	4979.8 ^A^	4886.1	4932.5	4874.9	30.165	0.002	0.671	0.257
Na ^6^	613.6	634.2	599.8	619.9	599.6	628.2	10.084	0.355	0.471	0.059
Mg ^7^	212.7 ^A^	183.0 ^B^	217.5 ^A^	207.6	199.3	206.3	3.592	0.000	0.499	0.096
Zn ^8^	54.9	38.5	53.3	39.4 ^b^	63.9 ^a^	43.4 ^b^	3.598	0.095	0.008	0.405
Fe ^9^	17.5	15.4 ^b^	18.1 ^a^	15.0 ^B^	17.0	19.0 ^A^	0.436	0.011	0.000	0.170

^1^ PHF—Polish Holstein-Friesian. ^2^ HH—Hereford. ^3^ LM—Limousin. ^4^ CH—Charolais. ^5^ K—Potassium. ^6^ Na—Sodium. ^7^ Mg—Magnesium. ^8^ Zn—Zinc. ^9^ Fe—iron. ^10^ SE—Standard error. Means followed by different superscript letters differ within rows (within the factor): A, B (*p* ≤ 0.01); a, b (*p* ≤ 0.05).

**Table 5:** Content of saturated fatty acids (%) in the longissimus thoracis muscle of crossbred bulls.

Fatty Acids	Crossbred Bulls (C)			Slaughter Age (Months) (SA)			SE ^7^	*p*-Value		
	PHF ^1^ × HH ^2^	PHF ^1^ × LM ^3^	PHF ^1^ × CH ^4^	15	18	21		C	SA	C × SA
IMF ^5^ (%)	1.58	1.34	1.49	1.07 ^b^	1.54	1.80 ^a^	0.125	0.697	0.045	0.118
∑SFAs ^6^	49.50	49.74	48.68	50.50 ^A^	49.56	47.83 ^B^	0.377	0.436	0.008	0.115
C 10:0 capric	0.045	0.047	0.041	0.050 ^a^	0.043	0.040 ^b^	0.002	0.185	0.018	0.847
C 12:0 lauric	0.061	0.069	0.059	0.071 ^a^	0.059 ^b^	0.059 ^b^	0.002	0.065	0.015	0.983
C 13:0 tridecanoic	0.011	0.013	0.010	0.013	0.011	0.011	0.001	0.443	0.546	0.405
C 14:0 iso	0.065	0.064	0.060	0.065	0.067	0.057	0.002	0.657	0.271	0.483
C 14:0 myristic	2.492	2.521	2.366	2.615	2.365	2.399	0.054	0.446	0.116	0.134
C 15:0 anteiso	0.210	0.209	0.188	0.219 ^a^	0.221 ^a^	0.167 ^b^	0.008	0.391	0.006	0.316
C 15:0 pentadecanoic	0.471	0.471	0.447	0.485 ^a^	0.487 ^a^	0.417 ^b^	0.013	0.644	0.033	0.252
C 16:0 iso	0.433	0.359	0.486	0.426	0.405	0.448	0.029	0.215	0.834	0.407
C 16:0 palmitic	25.72	26.24	25.17	26.13	25.29	25.72	0.255	0.229	0.406	0.274
C 17:0 margaric	1.033	1.049	1.013	1.088 ^a^	1.051	0.956 ^b^	0.020	0.744	0.022	0.248
C 18:0 stearic	18.77	18.49	18.65	19.18 ^a^	19.38 ^a^	17.35 ^b^	0.367	0.945	0.039	0.096
C 20:0 arachidic	0.131	0.145	0.129	0.136	0.128	0.141	0.004	0.183	0.424	0.217
C 22:0 behenic	0.053	0.064	0.058	0.061	0.055	0.058	0.003	0.335	0.704	0.055

^1^ PHF—Polish Holstein-Friesian. ^2^ HH—Hereford. ^3^ LM—Limousin. ^4^ CH—Charolais. ^5^ IMF—Intramuscular fat. ^6^ SFAs—Saturated fatty acids. ^7^ SE—Standard error. Means followed by different superscript letters differ within rows (within the factor): A, B (*p* ≤ 0.01); a, b (*p* ≤ 0.05).

**Table 6:** Content of unsaturated fatty acids (%) in the longissimus thoracis muscle of crossbred bulls.

Fatty Acids	Crossbred Bulls (C)			Slaughter Age (Months) (SA)			SE ^10^	*p*-Value		
	PHF ^1^ × HH ^2^	PHF ^1^ × LM ^3^	PHF ^1^ × CH ^4^	15	18	21		C	SA	C × SA
∑UFAs ^5^	50.50	50.26	51.32	49.48 ^B^	50.43	52.26 ^A^	0.380	0.443	0.007	0.115
∑MUFAs ^6^	43.76	43.53	43.85	42.52 ^b^	43.33	45.29 ^a^	0.409	0.939	0.011	0.052
C 14:1 myristoleic	0.476	0.541	0.546	0.569	0.453	0.540	0.028	0.507	0.203	0.152
C 16:1 palmitoleic	2.719	2.548	2.729	2.671	2.473	2.852	0.087	0.621	0.197	0.140
C 17:1 margaricoleic	0.619	0.595	0.683	0.648	0.601	0.646	0.022	0.237	0.611	0.155
C 18:1 T6+9 elaidic	0.523	0.470	0.480	0.479	0.523	0.471	0.019	0.485	0.477	0.344
C 18:1 T10+11 vaccenic	1.501	1.507	1.496	1.638	1.458	1.408	0.050	0.996	0.147	0.434
C 18:1 C9 oleic	35.56	35.51	35.35	34.17 ^B^	35.43	36.82 ^A^	0.370	0.965	0.009	0.055
C 18:1 C11	1.385	1.393	1.510	1.394	1.385	1.510	0.027	0.104	0.105	0.595
C 18:1 C12	0.195	0.183	0.204	0.195	0.202	0.184	0.006	0.386	0.481	0.636
C 18:1 C13	0.242	0.245	0.273	0.234	0.246	0.279	0.010	0.370	0.164	0.082
C 18:1 T16	0.376	0.361	0.363	0.363	0.385	0.351	0.008	0.720	0.237	0.461
C 20:1 gadoleic	0.168	0.178	0.177	0.157 ^b^	0.177	0.189 ^a^	0.005	0.679	0.045	0.344
∑PUFAs ^7^	6.74	6.73	7.47	6.96	7.10	6.97	0.288	0.496	0.976	0.306
C 18:2 linoleic	3.722	3.681	4.208	3.962	3.921	3.729	0.163	0.349	0.825	0.337
C 18:2 C9 T13	0.338	0.362	0.351	0.324	0.344	0.383	0.011	0.644	0.066	0.059
C 18:2 C9 T11 (CLA)	0.289	0.287	0.295	0.278	0.302	0.291	0.007	0.893	0.369	0.804
C 18:3 α-linolenic	0.905	0.947	0.980	0.941	0.978	0.913	0.035	0.679	0.746	0.144
C 20:2 eicosadienoic	0.038	0.076	0.047	0.042	0.041	0.079	0.011	0.309	0.241	0.257
C 20:4 arachidonic	0.851	0.784	1.004	0.833	0.911	0.895	0.062	0.340	0.862	0.357
C 20:5 eicosapentaenoic (EPA)	0.155	0.176	0.182	0.157	0.172	0.185	0.012	0.637	0.639	0.249
C 22:5 docosapentaenoic (DPA)	0.355	0.392	0.397	0.349	0.389	0.405	0.021	0.670	0.529	0.076
C 22:6 docosahexaenoic (DHA)	0.056	0.070	0.040	0.035	0.044	0.086	0.009	0.427	0.061	0.550
∑n-6 PUFAs ^8^	4.611	4.542	5.259	4.836	4.873	4.703	0.222	0.360	0.948	0.354
∑n-3 PUFAs ^9^	1.520	1.585	1.598	1.530	1.584	1.589	0.072	0.892	0.933	0.131

^1^ PHF—Polish Holstein-Friesian. ^2^ HH—Hereford. ^3^ LM—Limousin. ^4^ CH—Charolais. ^5^ UFAs—Unsaturated fatty acids. ^6^ MUFAs—Monounsaturated fatty acids. ^7^ PUFAs polyunsaturated fatty acids. ^8^ n-6 PUFAs ∑ (C 18:2 ∆9c,12c; C 20:2 ∆11c,14c; C 20:4 ∆5c,8c,11c,14c). ^9^ n-3 PUFAs ∑(C 18:3 ∆9c,12c,15c; C 20:5 ∆5c,8c,11c,14c,17c; C 22:5 ∆7c,10c,13c,16c,19c; C 22:6 ∆4c,7c,10c,13c,16c,19c).^10^ SE—standard error. Means followed by different superscript letters differ within rows (within the factor): A, B (*p* ≤ 0.01); a, b (*p* ≤ 0.05).

**Table 7:** Fatty acid ratios in the longissimus thoracis muscle of crossbred bulls.

Ratios	Crossbred Bulls (C)			Slaughter Age (Months) (SA)			SE ^8^	*p*-Value		
	PHF ^1^ × HH ^2^	PHF ^1^ × LM ^3^	PHF ^1^ × CH ^4^	15	18	21		C	SA	C × SA
MUFA ^5^/SFA ^6^	0.89	0.88	0.91	0.85 ^B^	0.88	0.95 ^A^	0.014	0.600	0.006	0.053
PUFA ^7^/SFA	0.14	0.14	0.16	0.139	0.145	0.147	0.006	0.416	0.892	0.355
n-6/n-3 PUFA	3.10	2.84 ^B^	3.30 ^A^	3.25 ^a^	3.08	2.92 ^b^	0.052	0.000	0.012	0.294

^1^ PHF—Polish Holstein-Friesian. ^2^ HH—Hereford. ^3^ LM—Limousin. ^4^ CH—Charolais. ^5^ MUFA—Monounsaturated fatty acids; ^6^ SFA—Saturated fatty acids. ^7^ PUFA—Polyunsaturated fatty acids; ^8^ SE—Standard error. Means followed by different superscript letters differ within rows (within the factor): A, B (*p* ≤ 0.01); a, b (*p* ≤ 0.05).
